# Hydrothermal conditioning of oleaginous yeast cells to enable recovery of lipids as potential drop-in fuel precursors

**DOI:** 10.1186/s13068-024-02561-x

**Published:** 2024-08-16

**Authors:** Shivali Banerjee, Bruce S. Dien, Vijay Singh

**Affiliations:** 1 Department of Agricultural and Biological Engineering, University of Illinois Urbana-Champaign, 1304 W. Pennsylvania Avenue, Urbana, IL 61801 USA; 2 Bioenergy Research Unit, National Center for Agricultural Utilization Research, USDA-ARS, Peoria, IL 61604 USA; 3 DOE Center for Advanced Bioenergy and Bioproducts Innovation, University of Illinois Urbana-Champaign, Urbana, IL 61801 USA

**Keywords:** Oleaginous yeast, Lipid recovery, Hydrothermal pretreatment, Cell lysis, Biofuels, Sustainability

## Abstract

**Background:**

Lipids produced using oleaginous yeast cells are an emerging feedstock to manufacture commercially valuable oleochemicals ranging from pharmaceuticals to lipid-derived biofuels. Production of biofuels using oleaginous yeast is a multistep procedure that requires yeast cultivation and harvesting, lipid recovery, and conversion of the lipids to biofuels. The quantitative recovery of the total intracellular lipid from the yeast cells is a critical step during the development of a bioprocess. Their rigid cell walls often make them resistant to lysis. The existing methods include mechanical, chemical, biological and thermochemical lysis of yeast cell walls followed by solvent extraction. In this study, an aqueous thermal pretreatment was explored as a method for lysing the cell wall of the oleaginous yeast *Rhodotorula toruloides* for lipid recovery.

**Results:**

Hydrothermal pretreatment for 60 min at 121 °C with a dry cell weight of 7% (w/v) in the yeast slurry led to a recovery of 84.6 ± 3.2% (w/w) of the total lipids when extracted with organic solvents. The conventional sonication and acid-assisted thermal cell lysis led to a lipid recovery yield of 99.8 ± 0.03% (w/w) and 109.5 ± 1.9% (w/w), respectively. The fatty acid profiles of the hydrothermally pretreated cells and freeze-dried control were similar, suggesting that the thermal lysis of the cells did not degrade the lipids.

**Conclusion:**

This work demonstrates that hydrothermal pretreatment of yeast cell slurry at 121 °C for 60 min is a robust and sustainable method for cell conditioning to extract intracellular microbial lipids for biofuel production and provides a baseline for further scale-up and process integration.

**Graphical abstract:**

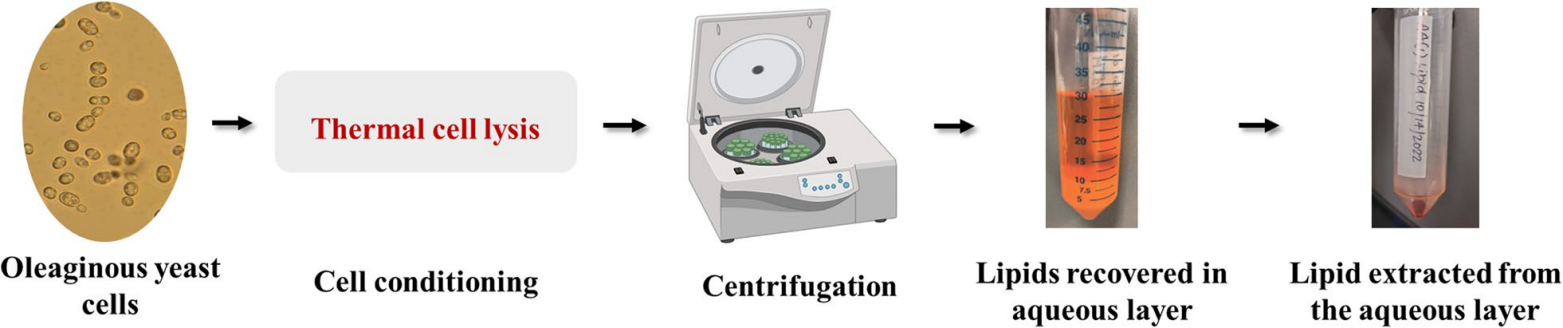

## Highlights

- Hydrothermal pretreatment was evaluated as a method for lysing oleaginous yeast cell walls.
- Thermal lysis of a yeast slurry (7% w/v, dry cell weight basis) at 121 °C for 60 min. followed by solvent extraction yielded 84.6 ± 3.2% (w/w) of the total lipids.
- Hydrothermal pretreatment of a yeast slurry (7% (w/v, dry cell weight basis)) at 170 °C for 10 min yielded 35.6 ± 0.6% (w/w) of the total lipids.
- Conditioning yeast slurry at 121 °C for 60 min is proposed to be a green scalable method for cell lysis.

## Introduction

Drop-in fuels are sustainable and renewable fuels that can be substituted directly (without engine modification) for either diesel [1], gasoline [2], or jet fuel [3]. Extensive research and development are ongoing to develop commercially viable drop-in fuels from biomass [4–8]. The present industrial routes to produce drop-in fuels are either to convert vegetable oil to green diesel or to ferment sugars to either ethanol or butanol and catalytically upgrade these to synthetic gasoline. The production of lipid-based biofuels is greatly influenced by the availability of lipids [9]. Single-cell (microbial) oil produced using lignocellulosic hydrolysates could provide an alternate feedstock for biofuel production [10–13]. These microbial lipids are obtained from a wide variety of microorganisms including yeast, bacteria, filamentous fungi, and microalgae that can accumulate lipids primarily in the form of triacylglycerols (TAGs) [14]. Oleaginous yeast allows for easier cultivation, higher cell densities, and better productivity than bacteria or fungi [15]. Oleaginous yeast cells can supplement plants as a source of oils because they have similar fatty acid profiles as those found in oil seeds. Furthermore, the use of agricultural resides (e.g., corn stover) or bioenergy crops grown on marginal farmlands are expected to increase oil production without impacting row cropland [16, 17]. Oleaginous yeast is defined as yeast that can accumulate at least 20% of their dry weight in lipids and many species of yeast under optimized culture conditions exceed 50% (w/w) lipid contents. Some of the genera containing oleaginous representatives are *Rhodotorula*, *Yarrowia*, *Lipomyces*, *Rhodosporidium, Cryptococcus*, *Candida*, and *Trichosporon* [15, 18–22]. Oleaginous yeast accumulates lipids from sugars when grown on nitrogen-limited media (high C/N ratio) [23–25]. The lipids in the yeast cells are stored as intracellular droplets and are used as an energy source, stress response, and cell growth [26].

Oil recovery methods developed for oilseeds do not work for yeast because of their tough cell walls. Therefore, cells need to be broken up first for solvent extraction of the lipids [27, 28]. A variety of methods have been proposed for the recovery of single-cell oil [29–32]. However, much of the cell lysis research has focused on microalgae, which may not be directly applicable to oleaginous yeast cells because of the differences in cell wall composition [30, 31, 33–36]. Notably, cell lysis research has also focused on the recovery of proteins and other high-value cell components. The conditioning/pretreatment/disruption of yeast cell biomass removes or weakens the protective cell walls to make the intracellular lipids more accessible to solvent extraction, facilitating high lipid recovery yields. Cell conditioning methods are broadly classified into chemical, physical, and enzymatic methods. The most applied techniques include treatment using microwaves, ultrasounds, shear abrasion, maceration, high-pressure homogenization, and hydrolysis (acidic, basic, or enzymatic) [37]. An ideal  cell conditioning step would enable efficient solvent extraction without degrading the TAGs, would be scalable and economical [38]. This includes the use of solvents that are compatible with current industrial practices. It is also preferable to avoid the need for drying the yeast [39]. When recovering lipids from wet cell biomass, the cell disruption method, lipid accessibility, mass transfer, and emulsion are the major factors that control the scalability, economics, and sustainability of a process [31, 40, 41]. While numerous laboratory-based methods have been proposed, there is still a need to develop new processes that are driven by engineering and economic targets. Methods for effective extraction of lipids from wet cell biomass are required for competitive process economics [42].

Most of the research on oleaginous yeasts and their industrial applications are centered around species like *Yarrowia lipolytica* or *Rhodotorula toruloides* due to their ease of genetic manipulation and ease of growth leading to an improved understanding of their physiology with a possibility of studying and engineering them further [43]. In similar lines, this work aims to evaluate the potential of thermal lysis of cell walls of the oleaginous yeast strain *Rhodotorula toruloides* as an aqueous process of lipid recovery. The process is analogous to the aqueous extraction of distillers corn oil in the corn dry grind ethanol process. The efficacy of the aqueous thermal process has been compared with the conventional cell conditioning methods including acid-assisted thermal lysis and sonication.

## Materials and methods

### Microbial biomass production

#### Pre-seed culture

The yeast strain *R. toruloides* Y-6987 was generously provided by the ARS Culture Collection (NCAUR, Peoria, IL) and was maintained on YPD (1% yeast extract, 2% peptone, 2% dextrose) agar plates and incubated at 28 °C for approximately 48 h. The colonies were transferred from the plates to culture tubes containing 3 mL of YPD incubated at 28 °C overnight and mixed at 250 rpm.

#### Seed culture

The culture tube pre-culture (1 mL) was transferred to a 250 mL baffled flask filled with 50 mL of YPD and incubated at 28 °C for approximately 18 to 24 h with 250 rpm shaking. The optical cell density (A_600_) of the pool seed culture was 30–36. The contents of the seed culture (~ 20 mL) were centrifuged and resuspended in sterile distilled water to an optical cell density of 50.

#### Fermentation

The concentrated pre-culture cells (A_600_) were reinoculated into the production flasks (500 mL) containing 100 mL of the lipid production media (per L: 3 g peptone, 8 g yeast extract, and 100 g glucose). The production flasks were incubated at 28 °C for 3 to 5 days with 250 rpm shaking. Sampling was done to monitor the glucose levels in the fermentation media and the cells were harvested when the concentration of glucose was less than 1%. The cells were harvested by centrifuging the culture in 250 mL bottles followed by the washing of the cell pellets using deionized water. The harvested cell paste was then stored at − 80 °C for further experiments on oil recovery.

### Determination of dry cell weight

To determine the dry weight, the oleaginous yeast cell paste/slurry (1 mL) was dried until a constant weight was achieved at 105 °C. The dry cell well was expressed in terms of grams of dry yeast per 100 mL of the cell paste/slurry.

### Estimation of total lipids in the microbial cells

The total lipids present in the cell biomass were estimated by a modified method [44]. Briefly, 5 mL of the cell slurry was mixed with 10 mL of isopropanol and 15 mL of hexane in a 50 mL screw top tube. The mixture was sonicated using a probe sonicator (Misonix XL-2000 Ultrasonic Liquid Processors) twice (for 1 min each) to disintegrate the microbial cell wall. Further, the slurry was shaken with a wrist action shaker (HB-1000 Hybridizer, UVP LLC, Upland, CA) for 10 min at room temperature. Then 16 ml of sodium sulfate solution (6.7%, w/v) was added and shaken for 10 min. The reaction mixture was centrifuged for 20 min at 5000 rpm, and the top phase was carefully transferred with a pipette to a pre-weighed screw-capped tube. The solvent was evaporated under a gentle stream of nitrogen and the lipid was weighed on an analytical balance.

To compare the accuracy of the method, a comparative estimation was made by extracting the lipids from the freeze-dried cell biomass using a conventional Soxhlet extractor. Briefly, 1 g of freeze-dried oleaginous yeast cells (in a Whatman filter paper bag) were subjected to Soxhlet extraction at 65–70 °C with hexane as the extracting solvent for 8 h. The microbial lipids selectively extracted into the hexane (150 mL) during the percolation process were recovered after the vaporization of solvent in a rotary evaporator. The yield of the recovered lipids was then calculated gravimetrically.

### Cell conditioning methods

**Fig. 1:**
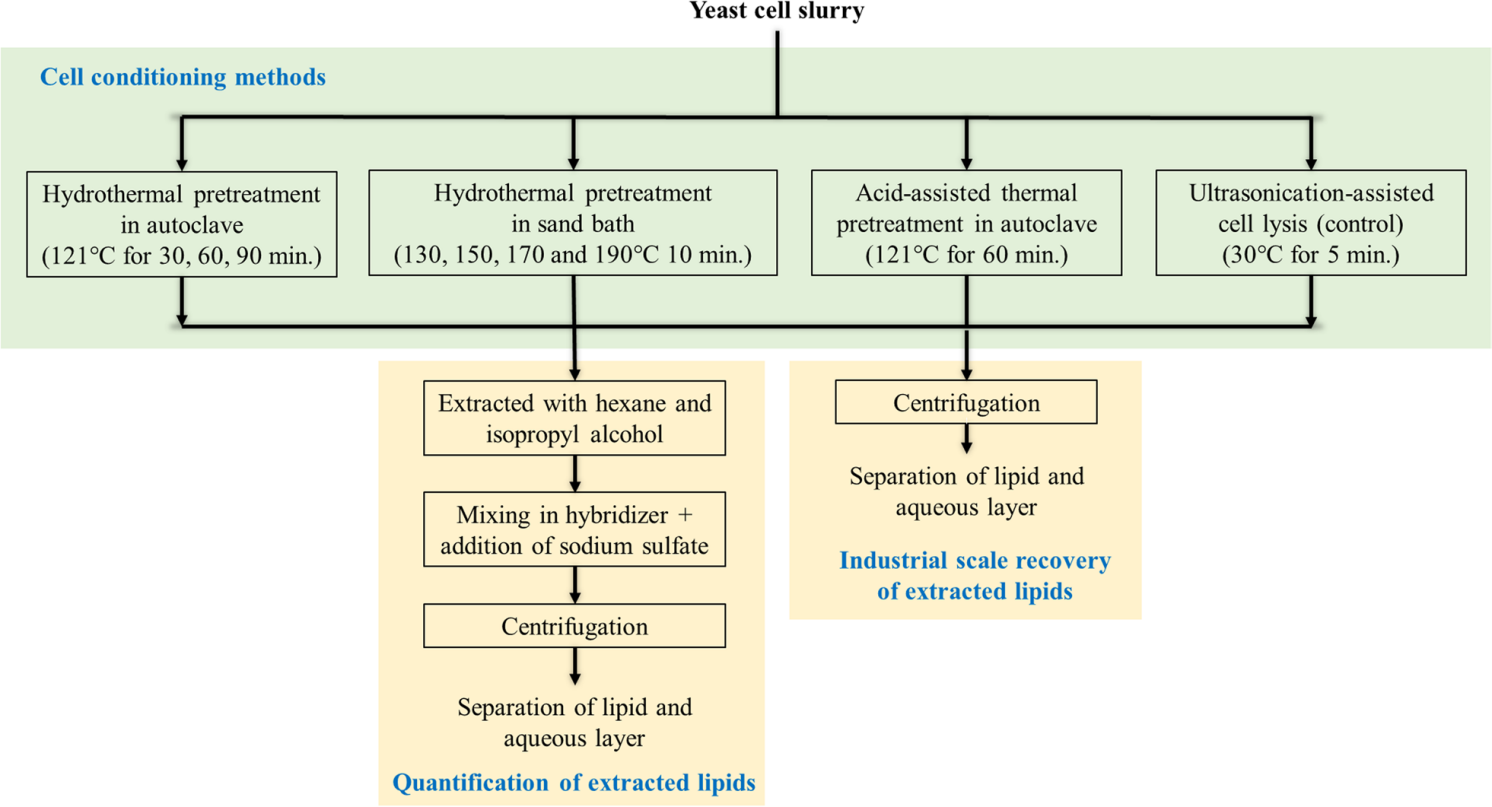
Flow diagram of the different conditioning strategies for lysing the oleaginous yeast cells followed by the recovery of extracted lipids

Figure 1 represents the schematic flow diagram of the different conditioning strategies for lysing the oleaginous yeast cells followed by the recovery of extracted lipids. A brief description of the cell conditioning methods is as follows:

#### Hydrothermal pretreatment in an autoclave

The microbial cell wall is known to disintegrate by autoclaving. This aspect was used in lysing the oleaginous yeast cells for recovering lipids. Autoclave was the choice of equipment to ascertain the scalability of the developed process. Briefly, 5 mL of oleaginous yeast slurry was loaded in autoclavable Schott bottles (20 mL). The dry cell weight in the slurry was ~14% (w/v) which was diluted with deionized water to achieve a dry cell weight of ~7% (w/v) to study the effect of solid loading on the recovery of lipids. The cell slurries were autoclaved at 121 °C for 30, 60, and 90 min. After cell conditioning, the reaction mixture was collected in a 50 mL screw top tube for extracting the lipids.

#### Acid-assisted hydrothermal pretreatment in an autoclave

Acid-assisted thermal lysis was carried out by loading 5 mL of yeast cell slurry (14%, w/v) in autoclavable Schott bottles (20 mL). The slurry was diluted using deionized water and hydrochloric acid (HCl) to make up a dry cell weight of 7% (w/v) and a total HCl concentration of 1% (v/v) in the final cell slurry mixture (10 mL). The reaction mixture was subjected to hydrothermal pretreatment in an autoclave at 121 °C for 60 min. The post-conditioning mixture was collected in a 50 mL screw top tube for extracting the lipids.

#### Sonication

Around 5 mL of the diluted cell slurry (7% w/v dry cell wt.) was taken in a 50 mL screw top tube and was subjected to sonication using a probe sonicator (Misonix XL-2000 Ultrasonic Liquid Processors) for 5 min. The lipids released after cell disruption were recovered and quantified.

#### Hydrothermal pretreatment in a sand bath

The wet microbial cell slurry (14%, w/v) was loaded in tube reactors and diluted with deionized water to achieve a dry cell weight of 7% (w/v). Hydrothermal pretreatment was carried out in a fluidized sand bath (IFB-51 Industrial Fluidized Bath, Techne Inc., Burlington, NJ, USA). The loaded reactors were immersed in the sand bath and heated to 130, 150, 170, and 190 °C with 10 min residence time. The time 10 min was chosen based on the previous studies conducted for the hydrothermal pretreatment of bioenergy crops [6, 45, 46] with the idea to subject the microbial cell slurry to hydrothermal pretreatment along with these lignocellulosic crops in an integrated biorefinery. A thermocouple (Penetration/Immersion Thermocouple Probe Mini Conn, McMaster-Carr, Robbinsville, NJ, USA) connected to a datalogger thermometer was used to measure the internal temperature of the tube reactor. After the residence time of 10 min, the autohydrolysis reaction was stopped by submerging the reactor vessel in cold water to rapidly decrease the temperature below 50 °C. The reaction mixture was collected in a 50 mL screw top tube for further recovery of the extracted lipids.

### Recovery of lipids

After the cell conditioning, the reaction mixture was collected in 50 mL screw top tubes followed by the addition of isopropyl alcohol (10 mL) and hexane (15 mL). The reaction mixture was shaken with a wrist action shaker (HB-1000 Hybridizer, UVP LLC, Upland, CA) for 10 min at room temperature. Then 16 mL of sodium sulfate solution (6.7%, w/v) was added and shaken for 10 min. The reaction mixture was centrifuged for 20 min at 5000 rpm, and the top phase was carefully transferred with a pipette to a pre-weighed screw-capped tube. The solvent was evaporated under a gentle stream of nitrogen and the recovered lipid was quantified gravimetrically.

The recoverable lipid content was calculated using Eq. (1):

1 $$\text{Lipid recovery efficiency }\left(\text{\%}\right)= \frac{W1}{W2}* 100,$$

where *w*_1_ is the lipid recovered by the pretreatment while w_2_ is the total lipid present in the whole yeast cell.

As depicted in the schematic flow diagram (Fig. 1), another strategy for recovering the extracted lipids at an industrially relevant scale includes centrifugation of the conditioned/pretreated cell slurry at 10,000 rpm for 20 min to separate the solid cell debris and the supernatant. The cell debris, supernatant, and emulsion were analyzed for the total lipid content in them.

### Fatty acid profile analysis

The extracted lipid samples were mixed with 2 mL of fresh hexane and 0.2 mL of 2 N KOH. The transesterification step was carried out according to the method previously published [47]. The samples were then run using the following conditions on the GC: injection volume = 1 μL; inlet = splitless mode; inlet temp = 240 °C; mobile phase = H_2_ with 7.5 psi; detector temp = 280 °C; H_2_ flow = 35 mL/min; airflow = 400 mL/min; oven temp = 140 °C, then ramp 15 °C/min to 240 °C, and hold for 2.5 min. The peaks were identified using two commercial reference standards (Nu-Chek Prep, product nos. 17A and 20A).

### Statistical analysis

All the experiments were conducted in triplicates and the results have been expressed as mean ± standard deviation. One-way analysis of variance (ANOVA) was conducted to determine the statistical significance of the response (*p* < 0.05). The difference in % lipid recovery by different pretreatment methods was evaluated using a one-way ANOVA. Means were compared using Tukey’s test at a 95% confidence interval. Two-way ANOVA with a general linear model was conducted to determine the statistical significance of pretreatment time (min) and the % dry cell weight of yeast slurry on % recovery of lipids (*p* < 0.05). Minitab Statistical software version 21 (Pennsylvania State University, USA) was used to analyze the data.

## Results and discussion

### Yeast lipid contents

**Fig. 2:**
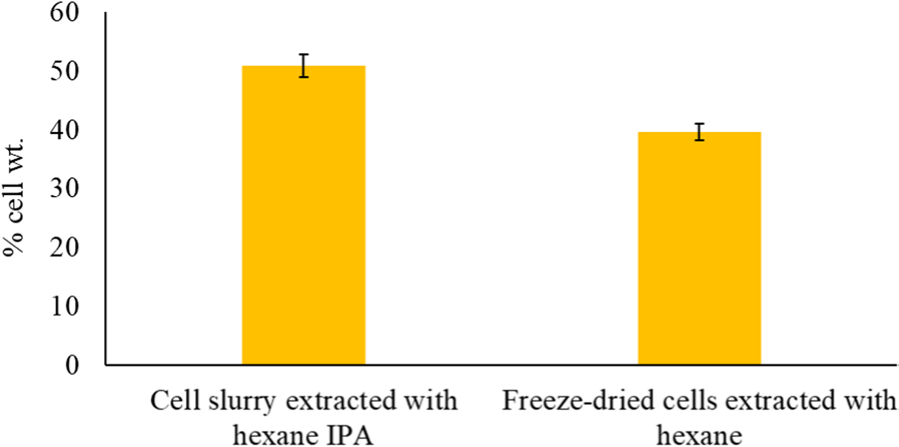
Lipid yields in % cell weight of *R. toruloides* extracted from wet cell slurry (with hexane and isopropyl alcohol) and freeze-dried cells (with hexane). The data represents the average of three independent experiments and the error bars indicate the standard deviation

Hexane and isopropyl alcohol were evaluated as potential co-solvents for measuring total lipid contents in wet cell slurries as an alternative to the conventional Bligh and Dyer method because it avoids the use of methanol and chloroform. Both methanol and chloroform are rated as undesirable and dangerous to use because of their health and environmental impacts [48]. The total lipid content of the *R. toruloides* cells was estimated to be 50.8 ± 1.9% (w/w) using ultrasound disruption followed by extraction with a solvent mixture of hexane and isopropyl alcohol (Fig. 2). However, the total lipid content (in the freeze-dried yeast cells) measured using the conventional Soxhlet extraction (with hexane as the extracting solvent) was 39.6 ± 1.4% (w/w). The difference in lipid contents can be attributed to the fact that the sonication of the oleaginous yeast cells (before extracting with hexane and isopropyl alcohol) disrupts the cell wall through the cavitation phenomenon, whereas the Soxhlet extraction works by diffusion without cell wall disruption [49, 50]. Further, isopropyl alcohol also allows for the recovery of polar (membrane) lipids versus the conventional Soxhlet method solely using hexane. In addition to lipids, the extractant includes carotenoids because of this red yeast. *R. toruloides* is a natural producer of carotenoids, including β-carotene, torulene, and torularhodin. This is desirable because these molecules are valued by the chemical, pharmaceutical, feed, and cosmetics industries [51, 52].

### Recovery of microbial lipids

The harvested yeast cells were concentrated in a 14.1 ± 0.1% (w/v) slurry based on dried weight. Two different concentrations of cell slurry were used during the conditioning procedures including ~14% and ~7% (w/v).

#### Hydrothermal pretreatment in an autoclave

**Fig. 3:**
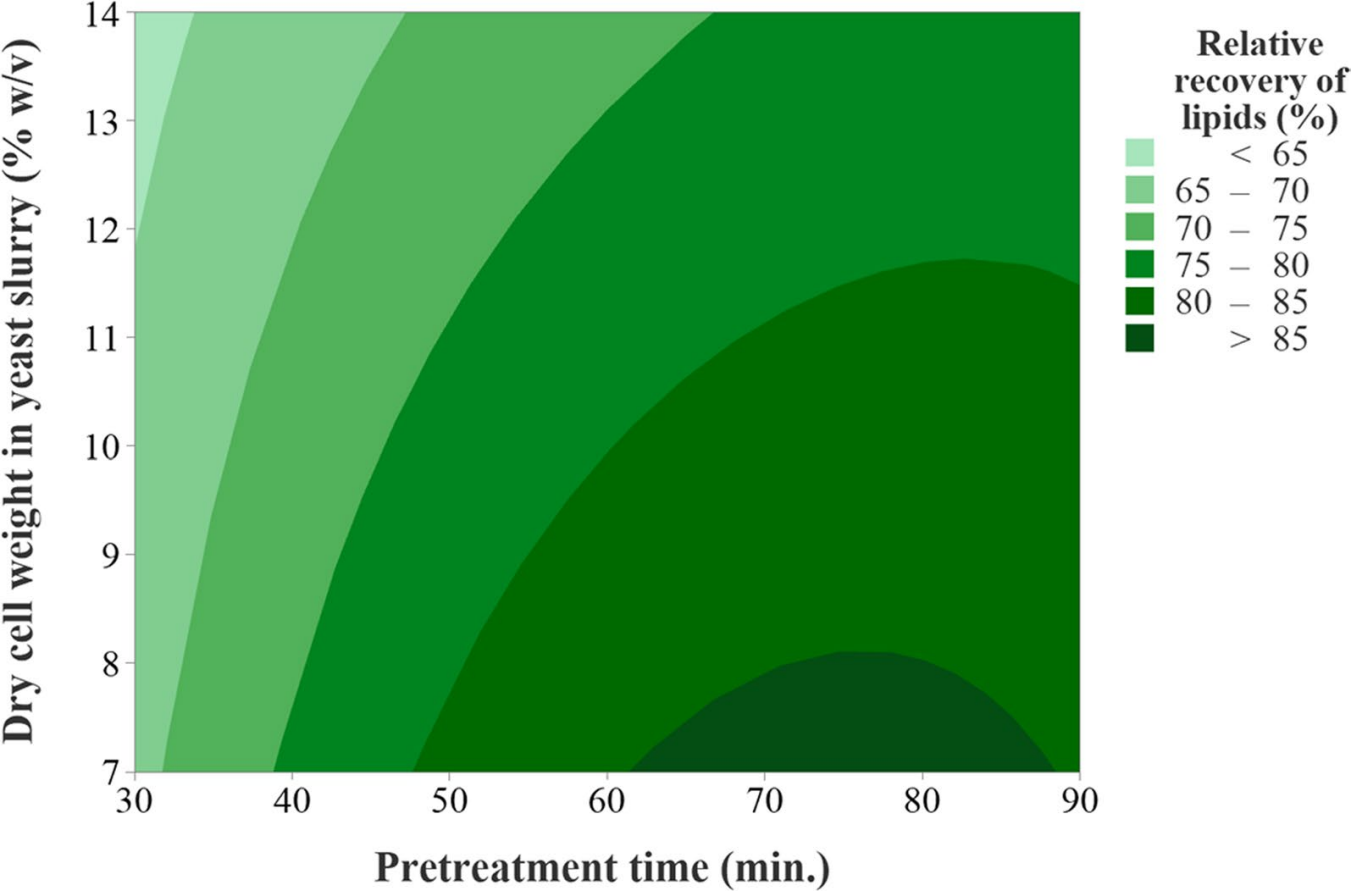
Contour plot showing the relative recovery of lipids from *R. toruloides* at different pretreatment time (min) and dry cell weight in yeast slurry (%, w/v) at 121 °C

While recovering lipids from oleaginous microbes, the scalability and economics of the process have been the key parameters. In particular, the thermal cell lysis in an autoclave was explored for recovering microbial lipids because it does not require adding chemicals or drying/freezing of the cells, both of which would add expense. Autoclaving is known to partially solubilize cell wall polysaccharides in hot water [53]. Preliminary results reveal that the hydrothermal pretreatment of the cell slurry (14%, w/v) at 121 °C for 30 to 90 min led to a lipid yield of greater than 63% w/w. The autoclave took 30–45 min to both reach the desired temperature and to cool down (to 60 °C). The statistical model contains two main effects, viz, pretreatment time (min) at 121 °C and dry cell concentration in yeast slurry (%) and their interaction. Both main effects were significant (*p* value < 0.05), but not their interaction (*p* value > 0.05) (Table 1). The linear model fitted the model well with *R*^2^ and adjusted *R*^2^ values of 94.9% and 92.8%, respectively. The maximum lipid recovery yield (~ 84%, w/w) obtained using a 7% (w/v) yeast slurry pretreated for 60 and 90 min at 121 °C was statistically similar (Table 2). The higher lipid recovery could be attributed to the higher severity of the pretreatment due to prolonged heating. The contour plot represents the % recovery of lipids as a function of yeast dry cell weight concentration in the slurry and pretreatment time (at 121 °C). It shows that the lipid recovery was greater than 80% (w/w) of the total lipids for a pretreatment time of 50 to 90 min and yeast cell slurry concentration of 7 to 11% (w/v) (Fig. 3).

**Table 1:** Analysis of variance (ANOVA) test for an experimental response for pretreatment time (min) and % dry cell concentration in yeast slurry for recovery of microbial lipids

Source	*DF*	Adj SS	Adj MS	*F* value	*p *value
Time (min)	2	803.92	401.961	81.60	< 0.0001
Dry cell concentration in yeast slurry (%)	1	276.59	276.587	56.15	< 0.0001
Time (min) * Dry cell concentration in yeast slurry (%)	2	26.34	13.172	2.67	0.110
Error	12	59.11	4.926		
Total	17	1165.96			

*R*^2^ = 94.9%; adjusted *R*^2^ = 92.8; predicted *R*^2^ = 88.6%

**Table 2:** Summary of average percent recovery of lipids from *R. toruloides* at different pretreatment times (min) and dry cell weight in yeast slurry (%, w/v) at 121 °C

Pretreatment time (min) at 121 °C	Dry cell wt. in yeast slurry (%, w/v)	Lipids recovered (g) per g of yeast cells (dry wt.)^*^	Relative recovery of lipids (%)^*#^
30	14	0.32 ± 0.01	63.3 ± 2.8^d^
30	7	0.35 ± 0.01	68.6 ± 0.9^c,d^
60	14	0.37 ± 0.01	73.6 ± 1.6^b,c^
60	7	0.43 ± 0.02	84.6 ± 3.2^a^
90	14	0.39 ± 0.01	77.4 ± 2.0^b^
90	7	0.43 ± 0.01	84.6 ± 2.0^a^

^*^Values represent mean ± standard deviation; ^#^values sharing the same alphabet (a, b, c, d) are not significantly different (*p* < 0.05)

#### Acid-assisted hydrothermal pretreatment in an autoclave

**Fig. 4:**
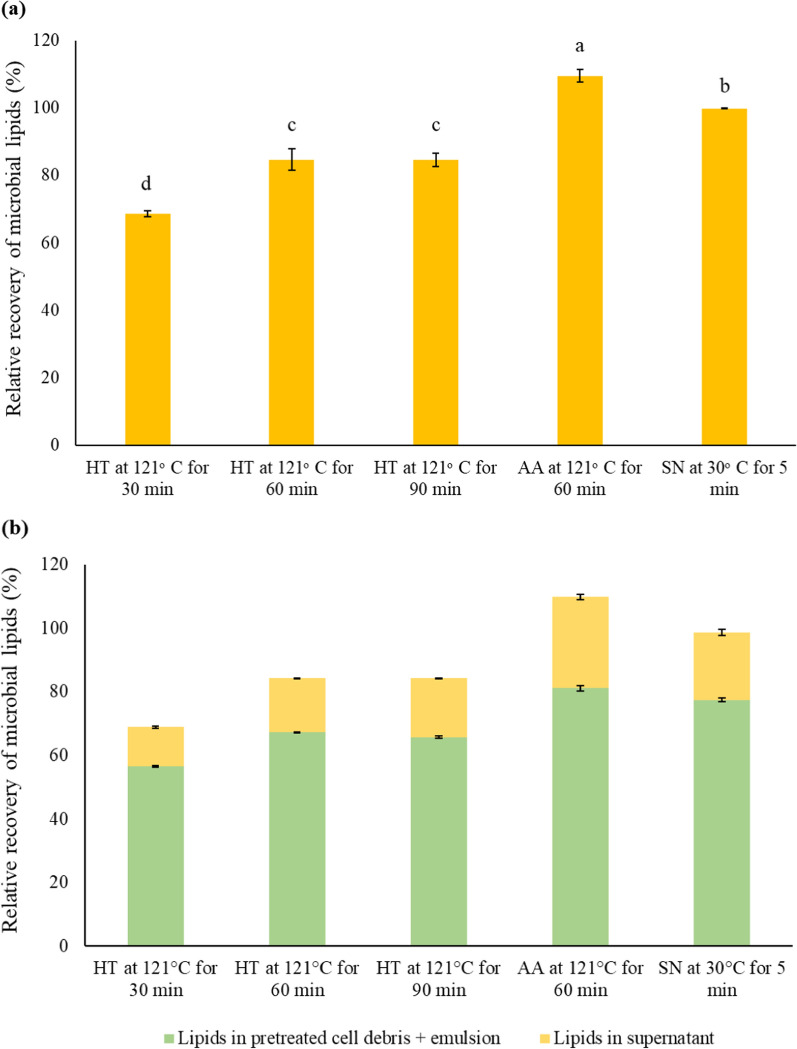
**a** Lipid recovery yields obtained after applying different cell lysis techniques to *R. toruloides* and **b** fractionation of the microbial lipids recovered from the pretreated solid cell debris + emulsion and the supernatant post-centrifugation. The data represent the average of three independent experiments, and the error bars indicate the standard deviation. The labels on the *x*-axis are abbreviated as *HT* hydrothermal, *AA* acid-assisted, *SN* sonication

The lipid yield at a yeast concentration of 7% w/v using acid-assisted hydrothermal pretreatment at 121 °C for 60 min is 109.5 ± 1.9% (w/w) (Fig. 4a). When heating was combined with an acid (1% v/v HCl), the thermal lysis of yeast cells led to greater cell wall disruption, which increased the recovery of intracellular lipids. Likely, acid hydrolyzes both the cell wall and protein layer protecting the lipid droplets. Xiao et al. reported that the hydrothermal pretreatment of *Phaffia rhodozyma* at 121 °C (0.1 Mpa, 15 min) with low acid concentration (HCl, 0.5 M) was found to be efficient in disrupting the cell wall leading to an astaxanthin extractability of 84.8 ± 3.2% [54]. Likewise treating *Candida* sp. LEB-M3 in an autoclave (at 121 °C, 101 kPa) for 15 min with HCl (2 M) also resulted in a very high lipid recovery (155.0 ± 4.1% lipids) [37]. It is notable that a higher lipid yield was observed than the actual microbial lipid content of the cells.

#### Sonication

The lipid recovery yields obtained with the sonication of the yeast slurry (7%, w/v) was 99.8 ± 0.03% (w/w) when extracted with hexane and isopropyl alcohol as the binary solvent system. Sonication works on the cavitation phenomenon wherein the bubbles formed during the rarefaction phase collapse at the compression phase, which gives a violent shock wave that generates pressure and heat and, ultimately, cell disruption. The lipid recovery from oleaginous microbes using sonication is affected by the time, temperature, cell concentration, frequency, power, and solvent system and has been studied in detail in the literature [50, 55–57]. In the present study, sonication at 30 °C for 5 min was evaluated as the conventional cell conditioning method for recovering total lipids from *R. toruloides* with hexane and isopropyl alcohol as co-solvents.

A lipid balance was performed on the disrupted yeast cells to determine how much of the lipids are recoverable in the liquid phase and the residual lipids in the disrupted yeast cells. The pretreated cell slurry was centrifuged to separate the cell debris from the supernatant to quantify the amount of lipids recovered in the liquid phase (Fig. 4b). The ideation of this sequential aqueous process of cell conditioning followed by centrifugation was adapted from the aqueous recovery of distillers corn oil in the corn dry grind ethanol process that has been commercialized [58, 59]. The hydrothermal pretreatment of the cell slurry at 121 °C for 30, 60, and 90 min led to a recovery of 18.1, 20.0, and 21.8%, respectively, of lipids in the supernatant after centrifugation. While 21.2 and 26.2% of the free lipids were recovered in the supernatant for the acid-assisted hydrothermal pretreatment and sonication-assisted pretreatment, respectively. The remaining 75–80% of the lipids were trapped in the residual cake of pretreated cells left after centrifugation and the emulsion.

#### Hydrothermal pretreatment in a sand bath

**Fig. 5:**
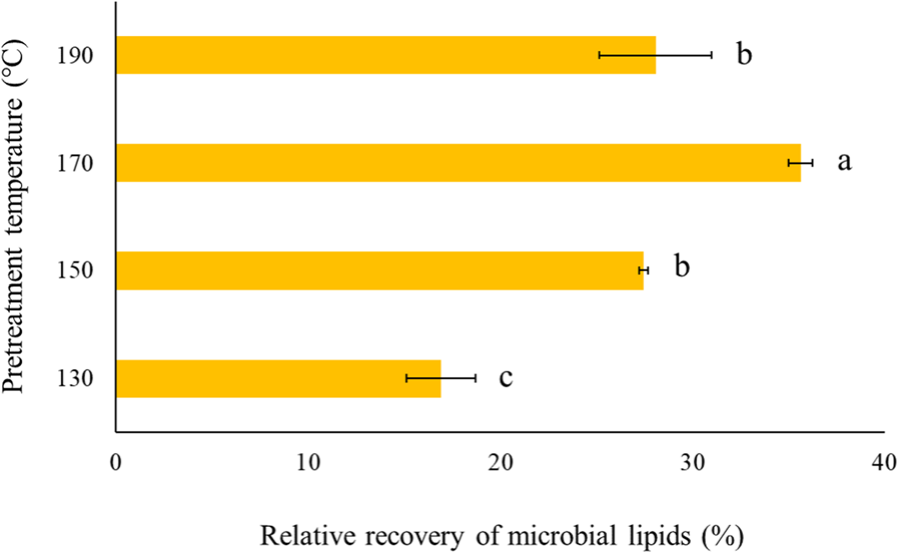
Lipid recovery yields obtained after applying hydrothermal pretreatment for 10 min. Each bar is the mean of three independent experiments, and the error bars indicate the standard deviation

The hydrothermal pretreatment of yeast cell slurry at elevated temperatures in a fluidized heat bath was used to evaluate the possibility of reducing the heating time by treating at higher temperatures. Lipids were recovered after hydrothermal pretreatment at 130 to 190 °C for 10 min (Fig. 5). The maximum lipid yield was only 35.6 ± 0.6% (w/w) of total lipids by pretreating the yeast slurry at 170 °C for 10 min. Heating at both 150 and 190 °C were similar to each other and significantly lower than 170 °C. The worst lipid yield (16.9 ± 1.8%, w/w) was measured when the yeast cells were treated at 130 °C for 10 min. In contrast, hydrothermal pretreatment at 121 °C for 60 min yielded 84.5 ± 3.2% (w/w) of total lipids. These results highlight the impact of pretreatment time on the extent of cell disruption and eventually the lipid yields. Kruger et al. studied the effect of thermal cell lysis on lipid recovery from oleaginous yeast at 170 and 220 °C [60]. The highest lipid recovery yield of 62.9% was obtained when the cells were subjected to thermal lysis at 220 °C for 60 min at a cell solid loading of 16%. The high-temperature pretreatment strategies (130–190 °C for 10 min) were tested so that an integrated system could be designed to pretreat the microbial lipid slurry along with the cellulosic biomass such as sugarcane, miscanthus, sorghum, and newer generation bioenergy crops that are being developed to accumulate lipids in the vegetative tissues [6, 45, 46, 61]. These wild-type and transgenic bioenergy crops could be cultivated on marginal lands and used to produce drop-in fuels that reduce dependency on fossil fuels and food crops [62]. Combined processing of the bioenergy crops and the microbial cell slurry would be a novel approach and would eventually improve the process economics by reducing the number of unit operations in an integrated biorefinery. However, the recovery of extracted microbial lipids and the separation of the pretreated bioenergy crops would be challenging and would need further research.

### Fatty acid profile

The dominant fatty acids synthesized by *R. toruloides* are palmitic acid (C16:0), stearic acid (C18:0), oleic acid (C18:1), and linoleic acid (C18:2) with other fatty acids accounting for < 15% of the total. The fatty acid profiles of the lipids recovered by various methods of cell conditioning are listed in Table 3. The palmitic, stearic, oleic, and linoleic acid contents in all the extracts are essentially the same, which suggests that the cell conditioning did not degrade the lipids. This is especially important for manufacturing biodiesel because the fuel properties are influenced by the fatty acid composition, including fatty acid chain length, saturation, and unsaturation. The ignition quality of biodiesel can be measured by cetane number, which increases for longer chain length of the fatty acids and decreases with increased branching [63, 64]. Polyunsaturated fatty acids reduce stability, cloud point, and cetane number and increase NOx emission, while the long-chain saturated fatty acids increase cloud point and cetane number, and improve oxidative stability with a significant reduction in NOx emission [65, 66]. The lipids recovered by each of the cell conditioning methods hold the potential to be converted into biodiesel due to their major fraction being oleic acid (C18:1) which is considered to be the most desirable fatty acid for biodiesel production [67].

**Table 3:** Fatty acid profile of lipids extracted from *R. toruloides* using different cell conditioning methods

Cell conditioning method	Fatty acid composition (%)									
	C14:0	C16:0	C16:1	C18:0	C18:1	C18:2	C18:3	C20:0	C22:0	Other
Sonication at 30 °C for 5 min	1.3	21.5	–	10.7	45.6	15.9	2.5	–	–	2.5
HT at 121 °C for 30 min	–	15.5	–	18.2	38.8	10.7	1.6	1.5	1.8	11.9
HT at 121 °C for 60 min	0.4	16.7	–	19.6	44.0	8.2	1.6	1.4	2.1	6
HT at 121 °C for 90 min	0.4	16.7	–	19.7	43.8	7.8	1.6	1.4	2.2	6.4
AA-HT at 121 °C for 60 min	1.0	30.2	0.3	10.5	47.2	5.9	0.9	0.4	0.8	2.8
HT at 130 °C for 10 min	1.3	22.5	–	12.5	43.5	14.7	2.4	–	0.6	2.5
HT at 150 °C for 10 min	1.7	17.8	–	10.6	46.9	15.9	4.5	–	–	2.6
HT at 170 °C for 10 min	–	17.7	–	11.3	46.4	16.9	–	–	1.8	6.2
HT at 190 °C for 10 min	1.7	17.1	–	10.2	47.2	16.8	4.8	–	–	2.2

*HT* hydrothermal, *AA-HT* acid-assisted hydrothermal

## Conclusion

The method selected for conditioning the cell slurry of *R. toruloides* for solvent extraction was critical for determining the final lipid yield. The maximum lipid yield (109.5 ± 1.5% (w/w)) was obtained for the acid-assisted hydrothermal pretreatment of yeast slurry at 121 °C for 60 min. That the recovery was greater than 100% indicates that this method was more efficient than the standard lipid recovery method. In the absence of acid, the hydrothermal pretreatment at 121 °C for 60 min led to a lipid recovery of 84.6 ± 3.2% (w/w) with organic solvents. In practice, the yeast cells can be concentrated into a slurry and heated using steam in a tank. However, the hydrothermal pretreatment of the cell slurry at 121 °C for 30, 60, and 90 min led to a recovery of 18.1, 20.0, and 21.8%, respectively, of lipids in the supernatant after centrifugation (can be recovered without using any organic solvent). The remaining 79–80% of the lipids were trapped in the residual cake of pretreated cells left after centrifugation and the emulsion. This is analogous to the aqueous recovery of lipids in the corn dry grind ethanol process that has been commercialized. The high lipid yield and the green nature of the process make it a promising method for lysing yeast cells and recovering lipids at an industrially relevant scale. The use of a mild temperature is critical because hydrothermal pretreatment of the cells at 170 °C for 10 min reduced the recovery to 35.6 ± 0.6% (w/w) of the total lipids. The highlighting feature of this method of cell conditioning is that it could be combined with the pretreatment of bioenergy crops in an integrated biorefinery. However, the recovery of extracted microbial lipids and the separation of the pretreated bioenergy crops would be challenging. The different cell conditioning methods render the extractable lipids intact, making them suitable for biofuel production. The extracted lipids contain a high fraction of palmitic, stearic, oleic, and linoleic acid chains which can be readily upgraded to green diesel fuel. Future research is needed to determine the composition of the residual yeast cell mass to determine its value (e.g., as a feed protein source). The end application of the cell residue could be decided after assessing the effect of temperature and acid on the residual proteins and carbohydrate fractions. Further, a detailed technoeconomic study is required to ascertain the applicability of the process at a commercial level.

## Data Availability

Data will be made available on request.
